# Integrated analysis of circulating tumour cells and circulating tumour DNA to detect minimal residual disease in hepatocellular carcinoma

**DOI:** 10.1002/ctm2.793

**Published:** 2022-04-05

**Authors:** Lina Zhao, Liping Jiang, Yunhe Liu, Xuebing Wang, Jinge Song, Yulin Sun, Yinlei Bai, Xiu Dong, Liying Sun, Jianxiong Wu, Yuchen Jiao, Xiaohang Zhao

**Affiliations:** ^1^ State Key Laboratory of Molecular Oncology National Cancer Center/National Clinical Research Center for Cancer/Cancer Hospital Chinese Academy of Medical Sciences and Peking Union Medical College Beijing China; ^2^ Department of Hepatobiliary Surgery National Cancer Center/National Clinical Research Center for Cancer/Cancer Hospital Chinese Academy of Medical Sciences and Peking Union Medical College Beijing China; ^3^ Department of General Surgery Beijing Friendship Hospital Capital Medical University Beijing China; ^4^ Liver Transplantation Center National Clinical Research Center for Digestive Diseases (NCRC‐DD) Beijing Friendship Hospital Capital Medical University Beijing China; ^5^ Jinchenjunchuang Clinical Laboratory Hangzhou China

Dear Editor,

Recurrence is the major reason for mortality after hepatectomy or liver transplantation surgery for hepatocellular carcinoma (HCC).^1^, ^2^, ^3^, ^4^ It is difficult to precisely manage adjuvant therapy to prevent recurrence after surgery. Here, we demonstrated that an integrated strategy of monitoring circulating tumour cells (CTCs) and circulating tumour DNA (ctDNA) could accurately detect minimal residual disease (MRD) and precisely predict recurrence in patients with HCC.^5^, ^6^, ^7^

A total of 80 patients with HCC were enrolled in this study, and 66 were eligible for analysis using postoperative serial blood samples (Figure 1A, Figure S1; Table S1). CTCs were positively selected by the asialoglycoprotein receptor using a microfluidic system and identified with pancytokeratins (Figure 1B). The first postoperative blood samples were analyzed for recurrence risk evaluation. Postoperative CTC positivity was significantly correlated with worse recurrence‐free survival (RFS) rates, with a sensitivity of 75% and specificity of 86.8% (*p <* .0001; hazard ratio [HR] 8.40, 95% confidence interval [CI] = 3.52–20.05) (Figure 2A, Table S2). To assess the ctDNA fraction, we first tested an approach using a personalized panel targeting mutations from whole‐exome sequencing (PPWES). Briefly, we performed WES on the tumour samples and selected ∼15 somatic mutations for each case (Tables S4, S5). A personalized assay was designed to profile the mutations in the matched ctDNA sample with mutation‐capsule technology (MCT).^8^, ^9^ ctDNA PPWES positivity, defined as one or more mutations detected in cfDNA, was strongly associated with a worse RFS rate (*p <* .0001; HR 11.77, 95% CI = 4.96–27.96), with 70.4% sensitivity and 93.8% specificity (Figure 2B, Table S2). The association between postoperative alpha‐fetoprotein (AFP) and des‐gamma‐carboxy prothrombin (DCP) positivity and poor RFS rates was significant (AFP: *p <* .0001; HR 4.77, 95% CI = 2.08–10.97, sensitivity: 28.6%, specificity: 94.7%; DCP: *p =* .0092; HR 2.76, 95% CI = 1.25–6.14, sensitivity: 32.1%; specificity: 86.8%) (Figure 2C,D; Table S2). When considering longitudinal surveillance, the median lead time from positive CTCs and ctDNA status by PPWES to recurrence detection by CT or MRI imaging was more than 3 months (Figures 1C,D and 3, Figure S2; Table S8).

**FIGURE 1:**
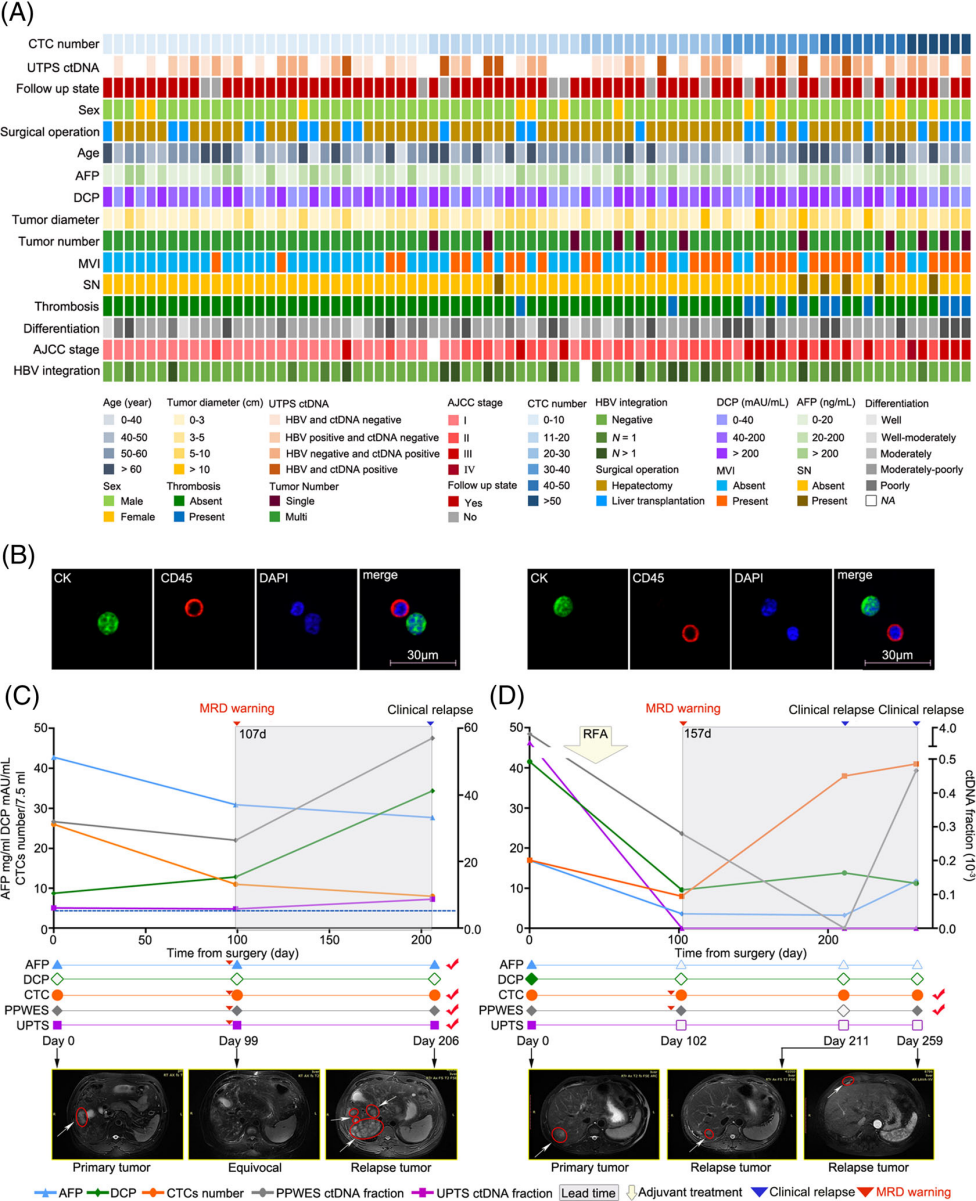
The basic ctDNA, CTC, tumour load and pathological characteristics of patients with hepatocellular carcinoma (HCC). (A) Heat map showing clinicopathologic, CTC and ctDNA UPTS detection data. Basic clinical information is indicated by the colored bar above the heat map. (B) CTCs were identified as pancytokeratins (Pan‐CK)^+^, CD45^–^ and DAPI^+^ cells at 600x magnification. The scale bar indicates 30 μm. The upper panels of (C) and (D) show the time‐course presentation of AFP, DCP, ctDNA and CTCs. MRD warning (red triangle) represents the recurrence prediction determined by the reminder of ctDNA (detected by PPWES or UPTS assays)/CTCs. Clinical relapse (blue triangle) represents disease recurrence confirmed by imaging diagnosis (CT/MRI/PET‐CT). Lead time was indicated by the interval between MRD warning and clinical relapse. The following adjuvant treatments were included: chemotherapy (CTx), radiofrequency ablation (RFA) and radiotherapy (RTx). In the middle panels of (C) and (D), the status of each circulating tumour marker at the time point of continuous follow‐up was identified. The solid icon represents the positive state, while the hollow icon represents the negative state. The circulating tumour marker tagged by a red check indicates an effective early warning for recurrence in this case. In the lower panels of (C) and (D), the imaging information from CT/MRI at different time points of each patient is presented. AFP, alpha‐fetoprotein; AJCC, American Joint Committee on Cancer; BCLC, Barcelona Clinic Liver Cancer; CT, computer tomography; CTCs, circulating tumour cells; ctDNA, circulating tumour DNA; DCP, des‐gamma‐carboxy prothrombin; MRD, minimal residual disease; MRI, magnetic resonance imaging; MVI, microvascular invasion; PET‐CT, positron emission tomography; PPWES, personalized panel targeting mutations from whole exome sequencing; UPTS, universal panel targeted sequencing

**FIGURE 2:**
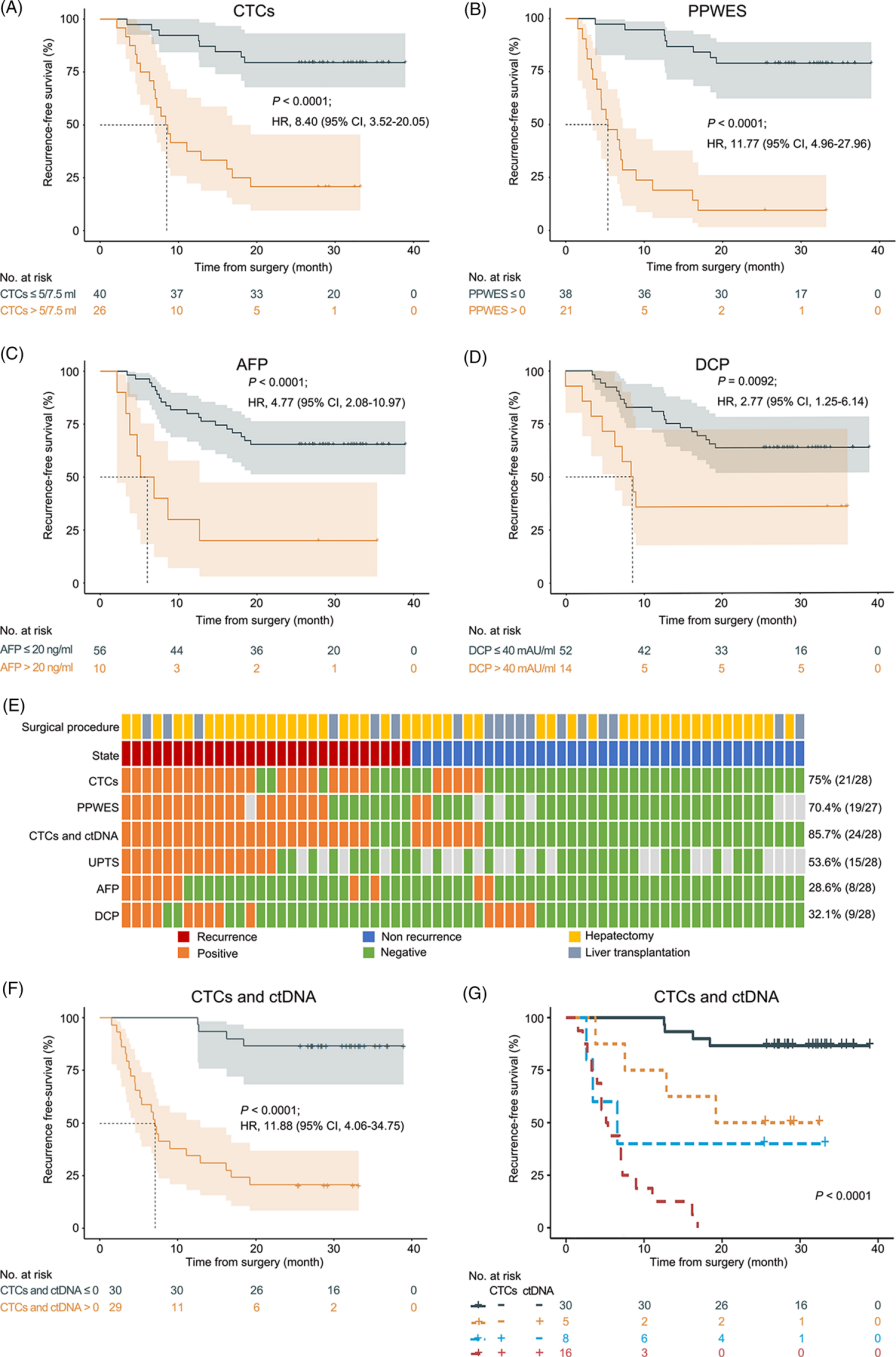
Prediction of recurrence‐free survival rates using CTCs, ctDNA, serum AFP and DCP in the first postoperative blood samples. Kaplan–Meier analysis based on (A) CTC enumeration, (B) ctDNA status by PPWES, (C) serum AFP, and (D) serum DCP. (E) Heat map shows the performance of CTC enumeration, ctDNA by PPWES, the combination of CTCs and ctDNA, ctDNA by UPTS, serum AFP and DCP in predicting the RFS rate using the first postoperative blood samples from HCC patients. (F) Kaplan–Meier analysis based on the combination of CTCs and ctDNA by PPWES. (G) Kaplan–Meier analysis of ctDNA^+^ /CTCs^+^, ctDNA^+^/CTCs^–^, ctDNA^–^/CTCs^+^ and ctDNA^–^/CTCs^–^ patients. The cutoff value, *p* values from log‐rank (Mantel–Cox) tests and HR values are indicated in the figure. The dashed lines in the figure represent the median survival (months). AFP, alpha‐fetoprotein; CI, confidence interval; CTCs, circulating tumour cells; ctDNA, circulating tumour DNA; DCP, des‐gamma‐carboxy prothrombin; HCC, hepatocellular carcinoma; HR, hazard ratio; PPWES, personalized panel targeting mutations from whole exome sequencing; RFS, recurrence‐free survival; UPTS, universal panel targeted sequencing

**FIGURE 3:**
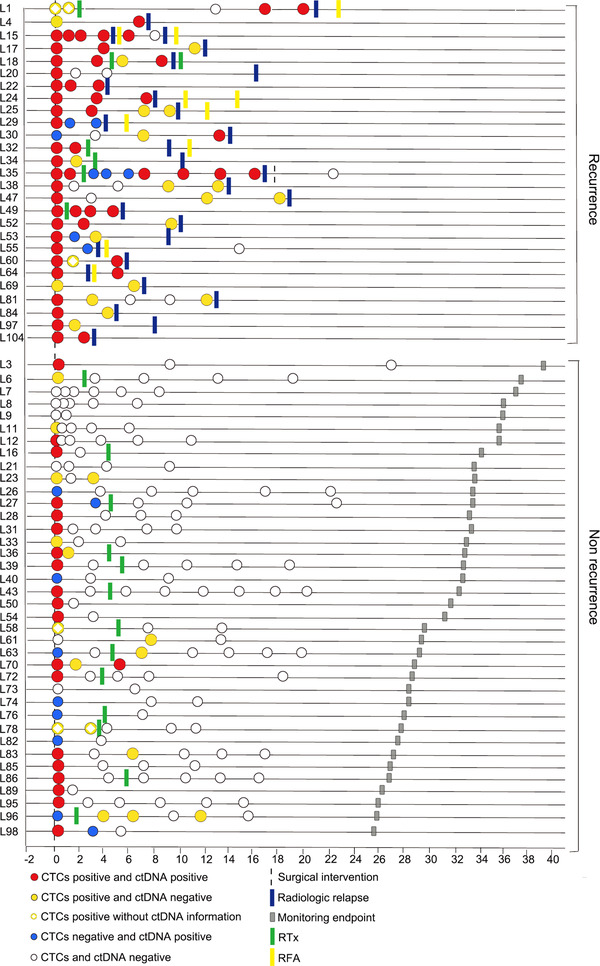
Longitudinal representation of CTC and ctDNA PPWES results for all samples from 66 patients. Patients with hepatocellular carcinoma (HCC) were divided into recurrence and non‐recurrence groups based on disease prognosis. Horizontal lines represent the disease courses of each HCC patient. Circle dots represent CTC and ctDNA PPWES status, including CTC and ctDNA positive (red dots), CTC positive and ctDNA negative (solid yellow dots), CTC positive without ctDNA information (hollow yellow dots), CTC negative and ctDNA positive (blue dots), CTC and ctDNA negative (white dots). The surgical intervention is shown by a vertical dashed line. Some of the patients also received adjuvant therapies, indicated by green (radiotherapy, RTx) and yellow (radiofrequency ablation, RFA) rectangles. The time points of disease recurrence were examined by imaging analysis, as indicated by blue rectangles. The monitoring endpoints of disease courses are presented as gray rectangles. CTCs, circulating tumour cells; ctDNA, circulating tumour DNA; PPWES, personalized panel targeting mutations from whole exome sequencing

Furthermore, we explored the synergistic effect of CTCs and ctDNA. The combination of CTCs and ctDNA exhibited enhanced sensitivity for predicting recurrence compared with analyzing CTCs or ctDNA alone (85.7% vs. 75% or 70.4%), with a specificity of 81.6% and the area under curve of .883 (Figure 2E, Figure S7). The combination provided a strong predictive value for the RFS rate, and RFS times in test‐negative patients were significantly greater than in test‐positive patients (HR 11.88, 95% CI = 4.06–34.75; *p <* .0001) (Figure 2F). The double‐negative (CTCs^–^/ctDNA^–^) patients had significantly better RFS times than single‐positive and double‐positive (CTCs^+^/ctDNA^+^) patients (median RFS for double negative, CTCs^+^/ctDNA^–^, CTCs^–^/ctDNA^+^ and double positive: not reached, 19.2, 6.57 and 5.25 months, respectively; *p <* .0001) (Figure 2G). A multivariate Cox regression analysis indicated that postoperative CTCs (*p = *.0223), ctDNA (*p *< .0001), AFP (*p =* .0021) and microvascular invasion (MVI) (*p *= .0403) were independent significant risk factors for HCC recurrence (Table S3).

Compared with CTC detection, the relatively complicated process and requirement for primary tumours could limit the clinical application of the PPWES strategy. In this case, we further analyzed other ctDNA‐based strategies with universal panel targeted sequencing (UPTS) and under a tumour‐naïve setup (Tables S6, S7). The MCT supports multiplex tests on the same ctDNA sample allowing a comparison of the performance of the strategy with PPWES in the same cohort cases (UPTS: *p <* .0001; HR 12.70, 95% CI = 4.94–32.64; PPWES: *p <* .0001; HR 12.62, 95% CI = 4.69–33.96) (Figure S3). The universal panel in this study covered the most frequent alterations in HCC, including the coding regions of *TP53/CTNNB1/AXIN1* and the promoter region of *TERT*. The full length of the hepatitis B virus (HBV) was also included to detect HBV integrations, and the breakpoint of the HBV‐human fusion sequence was used as a tumour‐specific biomarker. At least one genetic alteration in the panel was detected in 54 (74%) of the 73 tumours available. We applied MCT to profile the matched ctDNA sample.^8^ UPTS test‐positive status, defined by the detection of any tumour‐informed mutation or HBV integration in the postoperative ctDNA, was strongly associated with the RFS rate (*p <* .0001; HR 12.95, 95% CI = 5.08–33.03) (Figure 4A, Table S2).

**FIGURE 4:**
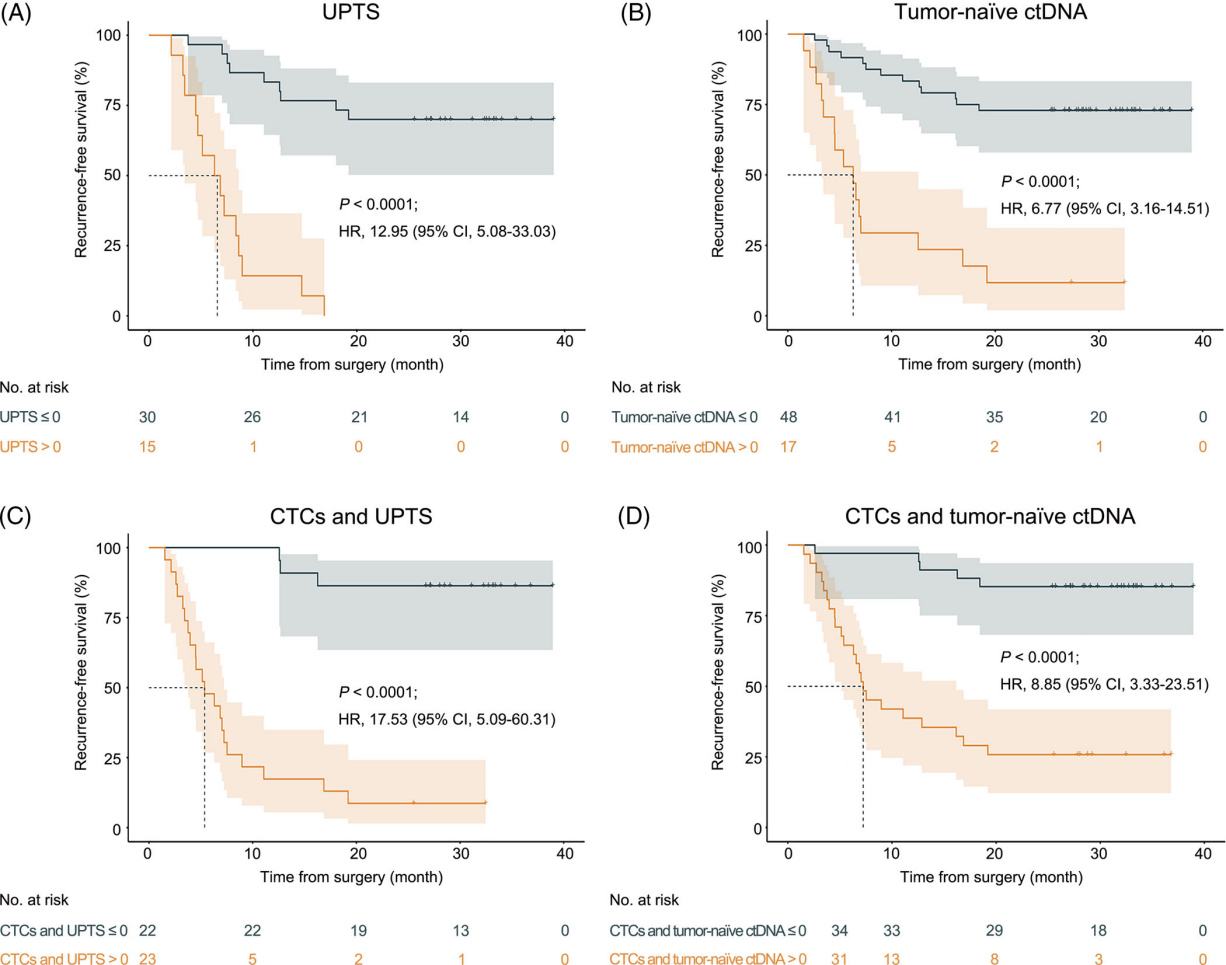
Prediction of recurrence‐free survival rates using ctDNA by the UPTS strategy and tumour‐naïve ctDNA strategy in the first postoperative plasma samples. Kaplan–Meier analysis based on (A) ctDNA by the UPTS strategy, (B) tumour‐naïve ctDNA, (C) the combination of CTCs and ctDNA by the UPTS strategy and (D) the combination of CTCs and tumour‐naïve ctDNA in predicting the RFS rate using the first postoperative blood samples. The cutoff value and *p* values were obtained using log‐rank (Mantel–Cox) tests, and HR values are indicated in the figure. The dashed lines in the figure represent the median survival (months). CI, confidence interval; CTCs, circulating tumour cells; ctDNA, circulating tumour DNA; HR, hazard ratio; RFS, recurrence‐free survival; UPTS, universal panel targeted sequencing

Additionally, we analyzed the performance of the tumour‐naïve strategy by profiling ctDNA without considering the mutation status in tumour tissue. We applied an algorithm trained with ctDNA samples from 65 HCC cases and 70 high‐risk individuals.^8^ Test‐positivity (score ≥ .5) by the tumour‐naïve approach was significantly associated with the RFS rate (*p <* .0001; HR 6.77, 95% CI = 3.16–14.51) (Figure 4B, Table S8). Although the significance was lower than that with PPWES or UPTS, this strategy was applicable to all 65 patients with postoperative blood samples. Furthermore, the combination of CTCs/UPTS and CTCs/tumour‐naïve assays both showed a strong association with the RFS rate (*p <* .0001; HR 17.53, 95% CI = 5.09–60.31; *p <* .0001; HR 11.88, 95% CI = 4.06–34.75) (Figure 4C,D).

MRD detection has been widely studied using different biomarkers including CTCs, ctDNA and different approaches. However, one previous study only profiled one type of biomarker and one approach; thus, it was difficult to compare the predictive value in different cohorts.^10^ Here, we profiled both CTCs and ctDNA in the same blood samples, and the ctDNA status was determined by three strategies with a personalized or universal panel in a tumour‐informed or tumour‐naïve setup. This comprehensive analysis enabled a head‐to‐head comparison of the biomarkers and approaches for predicting recurrence. Our study revealed that postoperative CTCs and ctDNA status were independent significant risk factors for HCC recurrence. The mutations of driving genes in CTC and ctDNA were highly consistent with tumour tissues (Figure S8, Table S9). The CTC status was complementary to the ctDNA status, and the combination provided improved detection of MRD and prediction of recurrence. In particular, the combination of the tumour‐naïve test was based on CTCs and ctDNA, which could be assessed through only one tube of blood with a universal protocol and showed good performance comparable to PPWES, the personalized and tumour‐informed assay, which was the most complicated and accurate. These findings underscore the importance of CTC and ctDNA integration in recurrence prediction and could also provide a reference for selecting strategies for HCC MRD surveillance.

## CONFLICT OF INTEREST

The authors declare that they have no conflict of interest.

## Supporting information

Supporting Information

Supporting Information
