# Supplementary material for: Integrated analysis of circulating tumour cells and circulating tumour DNA to detect minimal residual disease in hepatocellular carcinoma
Source: Clin Transl Med. 2022 Apr 5;12(4):e793. doi: 10.1002/ctm2.793 (PMC8982315; doi:10.1002/ctm2.793)
Supplement: Supplementary file 1 — Supporting Information [file CTM2-12-e793-s002.pdf]

Integrated analysis of circulating tumor cells and circulating tumor DNA to detect minimal residual disease in hepatocellular carcinoma

Lina Zhao,[1] Liping Jiang,[1] Yunhe Liu,[2] Xuebing Wang,[3,4] Jinge Song,[1] Yulin Sun,[1] Yinlei Bai,[5] Xiu Dong,[1] Liying Sun,[3,4] Jianxiong Wu,[2] Yuchen Jiao,[1] Xiaohang Zhao[1]

1. State Key Laboratory of Molecular Oncology, National Cancer Center/National Clinical Research Center for Cancer/Cancer Hospital, Chinese Academy of Medical Sciences and Peking Union Medical College, Beijing, China

2. Department of Hepatobiliary Surgery, National Cancer Center/National Clinical Research Center for Cancer/Cancer Hospital, Chinese Academy of Medical Sciences and Peking Union Medical College, Beijing, China

3. Department of General Surgery, Beijing Friendship Hospital, Capital Medical University, Beijing, China

4. Liver Transplantation Center, National Clinical Research Center for Digestive Diseases (NCRC-DD), Beijing Friendship Hospital, Capital Medical University, Beijing, China

5. Jinchenjunchuang Clinical Laboratory, Hangzhou, Zhejiang, China

Supplementary Information

Supplementary Materials and Methods

Supplementary Figures

Figure S1. Flow chart for patient selection in this study.

Figure S2. The Dynamic Changes of serum AFP, DCP, ctDNA and CTCs in plasma samples during HCC progression.

Figure S3. Head-to-head comparison of UPTS and PPWES strategies in predicting recurrence-free survival using first post-operative plasma samples.

Figure S4. Prediction of recurrence-free survival using serum CTCs and ctDNA in the hepatectomy plasma samples.

Figure S5. Prediction of recurrence-free survival using serum CTCs and ctDNA in the liver transplantation plasma samples.

Figure S6. Prediction of recurrence-free survival using serum CTCs and ctDNA in the early stage of HCC patients (AJCC stage = I and II) plasma samples.

Figure S7. The ROC curves of the serum CTC and ctDNA as a predictive model for HCC recurrence.

Figure S8. Mutation consistent among tumor tissue, ctDNA and CTCs.

Supplementary Tables

Table S1. Clinical and pathological characteristics of HCC patients and univariate analysis of recurrence

Table S2. Univariate Cox regression analyses of RFS in HCC patients

Table S3. Multivariate Cox regression analyses of RFS in HCC patients based on post-operative characteristics

Table S4. Tumor mutations detected in WES

Table S5. Personalized somatic mutations in tumor tissue and paired plasma by PPWES strategy

Table S6. Tumor mutations detected in UPTS strategy

Table S7. ctDNA mutations detected in UPTS strategy with paired tumor tissue

Table S8. The comprehensive information of each timepoint of HCC patient in cohort

Table S9. Concordance of mutation profiles of CTCs and matched ctDNA detected in UPTS strategy

Supplementary Materials and Methods

Study population

A total of 80 HCC patients who underwent surgical therapy were enrolled in the study between February 2017 and June 2020 at the Cancer Hospital of Chinese Academy of Medical Sciences and the Beijing Friendship Hospital of Capital Medical University. The inclusion criteria were as follows: (a) pathologic or radiographic diagnosis of HCC; and (b) surgical treatment by either hepatectomy or liver transplantation. The clinical information of patients with HCC was collected, such as age, sex, imaging data, pathological results and so on. Tumor stage was determined according to the AJCC and the Barcelona Clinic Liver Cancer (BCLC) staging systems. Tumor differentiation was defined according to the Edmondson grading system. Nine healthy controls, five patients with benign liver disease and three non-liver malignant cancer patients were enrolled as negative control. This study approved by the Institutional Review Board of the Ethics Committee of Cancer Hospital of Chinese Academy of Medical Sciences (ID: NCC3094) and Beijing Friendship Hospital (ID: 1018-768). All the doner liver came from legal organ donors. An informed consent was obtained from all subjects and the study was performed in accordance with the guidelines of the Declaration of Helsinki.

Patient characteristics

A total of 80 patients with HCC were enrolled continuously, 58 HCC patients underwent hepatectomy were enrolled from 2017/2/8 to 2018/8/27, 22 HCC patients underwent liver transplantation were enrolled from 2017/8/12 to 2018/8/3. The clinicopathological characteristics of 80 evaluable patients were summarized in Table S1 and Figure S2A, including 68 males and 12 females with a median age of 54 (range, 34 - 77), 87.5% (70/80) of hepatitis B virus (HBV) and 6.25% (5/80) of hepatitis C virus (HCV) positive. Patients underwent hepatectomy (N = 58) or liver transplantation (N = 22) with a median follow-up of 26.1 months (range, 19.3-42.6 months) after surgical therapy, and 25 patients were received additionally adjuvant

therapy (radiotherapy or radiofrequency ablation). During follow-up, 34 patients (42.5%) experienced disease recurrence confirmed by the imaging diagnosis, 27 patients went through hepatectomy with recurrence rate of 46.6% (27/58), while 7 patients accepted liver transplantation with recurrence rate of 31.8% (7/22). Clinical samples including 73 liver tumor tissues and 263 blood samples before and after surgery (every 3 months for first year and every 6 months afterward) were systematically collected from the 80 HCC patients, 81.3% (65/80) of them have collected peripheral blood sample at least 2 times and 60% (48/80) of them have collected peripheral blood sample at least 3 times. Before surgical therapy, the levels of circulating markers of patients, such as CTCs, ctDNA, AFP and DCP were assessed first. After hepatectomy or liver transplantation, circulating biomarkers such as CTCs, ctDNA, AFP and DCP, and imaging analysis of MRI, CT or positron emission tomography (PET-CT) were used for dynamic disease monitoring.

CTCs enrichment and identification

CTCs enrichment was performed using a CytoQuest^{CR} microfluidic system (ABNOVA). Buffy coats were rinsed with RPMI medium and gently injected into an asialoglycoprotein receptor (ASGPR)-coated microfluidic slide at room temperature (CAT: KA4573, ABNOVA).[1-4] After the slide was fixed and ventilated, the captured cells were immunostained with an antibody cocktail containing anti-pan-cytokeratins (PanCK, CK8/18/19) antibody conjugated to fluorescein isothiocyanate (FITC), anti-CD45 antibody conjugated with phycoerythrin (PE) (CAT: KA4573, ABNOVA), and 2-(4-Amidinophenyl)-6-indolecarbamidine dihydrochloride (DAPI). Stained cells were imaged using a fluorescence microscope (Nikon). CTCs were defined as PanCK (+), CD45 (-), and DAPI (+), whereas white blood cells (WBCs) were defined as PanCK (-), CD45 (+), and DAPI (+) cells.

The CTCs were captured and enumerated in dynamically in 66 patients. Then, the level of CTCs to distinguish patients with recurrence of disease from those with no recurrence in 2 years was further explored. The thresholds of 1 to 20 cells were

systematically correlated with progression-free survival in preoperative and first postoperative sample. The median recurrence-free survival among patients with levels above or below each threshold differed at the level of 1 CTC/7.5 ml and reached a plateau at > 5 CTCs/7.5 ml. Meanwhile, the Cox proportional-hazards ratio could stratify the difference between recurrence and non-recurrence of HCC, and reached a plateau. Thus, CTCs positivity was defined as CTCs count of > 5/7.5 ml. While the captured CTCs were rare in non-HCC control group (median: 0/7.5 ml, range:0-2, N = 19). [5, 6]

Determination of serum AFP and DCP concentrations

Serum AFP and des-gamma-carboxy prothrombin (DCP) levels were determined using ARCHITECT PIVKA-II and ARCHITECT AFP Reagent Kits (Abbott Laboratories, Chicago, IL, USA) and an Abbott ARCHITECT i2000SR Chemical Luminescence Immunity Analyzer (Abbott Laboratories).

DNA extraction and library preparation

DNA from WBCs and frozen tumor samples was extracted using QIAamp DNA Tissue & Blood kits (QIAGEN). Formalin-fixed, paraffin-embedded (FFPE) tissues and haematoxylin and eosin (H&E) sections were retrieved from the pathology department and reviewed by a consultant histopathologist. Qiagen GeneRead kits (QIAGEN) were used to extract DNA from FFPE tissues. Cell-free DNA was extracted from 3-6 ml of plasma using Apostle MiniMax cfDNA isolation kits (Apostle; Sunnyvale, CA, USA). Extracted DNA was quantified using a Qubit dsDNA HS Assay (Thermo Fisher Scientific). Tumor and matched WBC DNA libraries were prepared using KAPA Hyper Prep kits (Roche). The ctDNA library preparation was performed using previously-described the Mutation Capsule technology.[7]

Targeted sequencing, tumor exome sequencing and bioinformatics analysis

Whole genome libraries of tumors and matched WBC DNA was enriched by

hybridisation and analysed using a universal panel covered the most frequent alterations in HCC for targeted sequencing. Agilent SureSelectXT Human All Exon V6 probes and reagents were used for capturing exonic regions of each library. (Agilent; Santa Clara, CA, USA). Exome libraries were pooled and sequenced on a NovaSeq 6000 (Illumina; San Diego, CA, USA) with 150-bp paired-end sequencing to a median depth of 181× for tumor tissue samples and 190× for WBC samples (exome sequencing) and 1175× for tumor tissue samples (targeted sequencing after removing duplicate molecules). We removed residual adapters from sequencing raw data (FASTQ file) and low-quality reads with Trimmomatic (v0.36). Clean reads were mapped to the UCSC human reference genome hg19 and the HBV genome using Burrows-Wheeler aligner software (BWA, v 0.7.15). Basic processing, duplicate marking, local realignments, and score recalibration were performed using the Genome Analysis Toolkit (GATK, v3.6), Picard (v2.7.1), and Samtools (v1.3.1). Candidate somatic mutations were detected by comparing the sequencing data from tumor tissue samples using MuTect1 and Strelka software. The criteria adopted for defining mutations in tumors were allele fraction ≥1% and total reads ≥ 4. All selected mutations were validated by manual inspection using the Integrated Genome Viewer (IGV).[(8)]

ctDNA by PPWES and bioinformatics analysis

Whole genome libraries of ctDNA with customised adaptors were used as templates to profile mutations identified in the matched tumor tissue. The customised adaptors contained sufficient unique DNA barcodes to identify each original molecule.[(7)] For each patient, ~15 (up to 20) tumor-specific variants from the whole exome sequencing (WES) results were selected for primer design (Tables S4, S5) based on the confidence and frequency of the mutation. Oligo software (v7.53) was used to design multiple PCR primer pairs for the two rounds of nested amplification. Primer uniqueness in the human genome (http://genome.ucsc.edu/) was verified to ensure amplification efficiency. The targeted sequencing libraries of tumor-specific variants were sequenced

on an Illumina Novaseq 6000 with a median depth of 13417× after removing duplicate molecules. SNV calling and annotation of candidate variations were performed as previously described.[7]

The ctDNA/cfDNA ratio model

The ctDNA/cfDNA ratio model was performed using R statistical environment version 3.6.3. The model was developed to estimate the ctDNA fraction based on allele frequency and sequencing depth of somatic mutations in tumor tissue and paired plasma samples, which was described in the previous study. [9] It was based on the following assumptions: (a). Because of the low fraction of ctDNA, there is a chance that some mutations present in the tumor tissue were not detected in the corresponding plasma sample. The algorithm is also based on the assumption that this tumor-plasma mismatches results from the low concentration of mutant molecules and/or randomness in sampling. (b). The estimation model assumes that mutant allele reads, and non-mutant wild-type allele reads fit well with a binomial distribution. To evaluate the biological noise of random mutations in patient plasma samples, we determined the fraction of 20 mutations that were not detected in matched tumor samples. To achieve 100% specificity, customised primers from more than five patients were pooled for cross-validation and were used to exclude non-specific noise. For example, a tumor-specific T > C mutation of gene A was identified in the matched plasma sample, and this variant was identified in another plasma sample, but it was not detected in the corresponding tumor. We determined that if the alteration numbers of the tumor-specific T > C mutation of gene A in the matched plasma was at least 2-fold that of another non-tumor-specific plasma, the mutation was regarded as true. The ctDNA PPWES positive patients were defined as ctDNA fraction > 0.

ctDNA by UPTS and bioinformatics analysis

Whole genome libraries of ctDNA were enriched for the target regions using previously described mutation capsule technology.[7] We profiled the following target

regions in ctDNA: (a) the coding regions of *TP53*, *CTNNB1*, *AXIN1*, and the *TERT* promoter region; and (b) HBV integrations.[(10, 11)] The enriched and amplified libraries were sequenced on an Illumina HiSeqX Ten instrument with 150-bp paired-end sequencing to a median depth of 6140× for ctDNA after removing duplicate molecules. Sequencing reads were processed using self-developed program to extract tags and remove sequence adapters.[7] We removed residual adapters and low-quality reads with Trimmomatic (v0.36). Clean reads which were mapped to the human reference genome hg19 and HBV genome with BWA (v0.7.15).[10] SNV/indels in the targeted regions were called using samtools mpileup (v 0.1.1722).[12] The criteria adopted for defining mutations in ctDNA were an allele fraction ≥ 0.03%. HBV integrations were identified by Crest, and at least four soft-clip read supports were needed.[13] To ensure accuracy, reads with the same tags, start, and end coordinates were grouped into unique identifier families (UID families). UID families containing at least two reads, in which at least 80% of reads were of the same type were defined as effective unique identifier families (EUID families). Each mutation frequency was calculated by dividing the number of alternative EUID families by the sum of the alternative and reference frequencies. The mutations were manually reviewed using IGV. Candidate variations were annotated using the Ensembl Variant Effect Predictor (VEP).[13]

Statistical analysis

Statistical analysis was conducted using R software (Version 3.6.1). The correlations between circulating tumor markers and clinical parameters were calculated and visualized using the “ggplot2”, “Hmisc” and “corrplot” R packages. The heatmaps were visualized by “pheatmap”. The “Survival” and “survminer” were used to perform univariate and multivariate analysis of the Cox regression model and for visualization. Continuous parameters were dichotomized for RFS, which was generated by Kaplan-Meier method and compared by using Log-rank test. All statistical tests were two-tailed and were considered significant at $P < 0.05$.

References

1. Shi B, Abrams M, Sepp-Lorenzino L. Expression of asialoglycoprotein receptor 1 in human hepatocellular carcinoma. The journal of histochemistry and cytochemistry : official journal of the Histochemistry Society. 2013;61(12):901-9.
2. Mu H, Lin KX, Zhao H, Xing S, Li C, Liu F, et al. Identification of biomarkers for hepatocellular carcinoma by semiquantitative immunocytochemistry. World journal of gastroenterology. 2014;20(19):5826-38.
3. Zhang Y, Zhang X, Zhang J, Sun B, Zheng L, Li J, et al. Microfluidic chip for isolation of viable circulating tumor cells of hepatocellular carcinoma for their culture and drug sensitivity assay. Cancer biology & therapy. 2016;17(11):1177-87.
4. Court CM, Hou S, Winograd P, Segel NH, Li QW, Zhu Y, et al. A novel multimarker assay for the phenotypic profiling of circulating tumor cells in hepatocellular carcinoma. Liver transplantation : official publication of the American Association for the Study of Liver Diseases and the International Liver Transplantation Society. 2018;24(7):946-60.
5. Cristofanilli M, Budd GT, Ellis MJ, Stopeck A, Matera J, Miller MC, et al. Circulating tumor cells, disease progression, and survival in metastatic breast cancer. The New England journal of medicine. 2004;351(8):781-91.
6. Galardi F, De Luca F, Biagioni C, Migliaccio I, Curigliano G, Minisini AM, et al. Circulating tumor cells and palbociclib treatment in patients with ER-positive, HER2-negative advanced breast cancer: results from a translational sub-study of the TREnd trial. Breast cancer research : BCR. 2021;23(1):38.
7. Qu C, Wang Y, Wang P, Chen K, Wang M, Zeng H, et al. Detection of early-stage hepatocellular carcinoma in asymptomatic HBsAg-seropositive individuals by liquid biopsy. Proceedings of the National Academy of Sciences of the United States of America. 2019.
8. Fujikura K, Hosoda W, Felsenstein M, Song Q, Reiter JG, Zheng L, et al. Multiregion whole-exome sequencing of intraductal papillary mucinous neoplasms reveals frequent somatic KLF4 mutations predominantly in low-grade regions. Gut. 2021;70(5):928-39.
9. Zhao D, Yue P, Wang T, Wang P, Song Q, Wang J, et al. Personalized analysis of minimal residual cancer cells in peritoneal lavage fluid predicts peritoneal dissemination of gastric cancer. Journal of hematology & oncology. 2021;14(1):164.
10. Zhang W, He H, Zang M, Wu Q, Zhao H, Lu LL, et al. Genetic Features of Aflatoxin-Associated Hepatocellular Carcinoma. Gastroenterology. 2017;153(1):249-62 e2.
11. Sung WK, Zheng H, Li S, Chen R, Liu X, Li Y, et al. Genome-wide survey of recurrent HBV integration in hepatocellular carcinoma. Nature genetics. 2012;44(7):765-9.
12. Li H, Handsaker B, Wysoker A, Fennell T, Ruan J, Homer N, et al. The Sequence Alignment/Map format and SAMtools. Bioinformatics. 2009;25(16):2078-9.
13. McLaren W, Gil L, Hunt SE, Riat HS, Ritchie GR, Thormann A, et al. The Ensembl Variant Effect Predictor. Genome biology. 2016;17(1):122.

Supplementary Figures

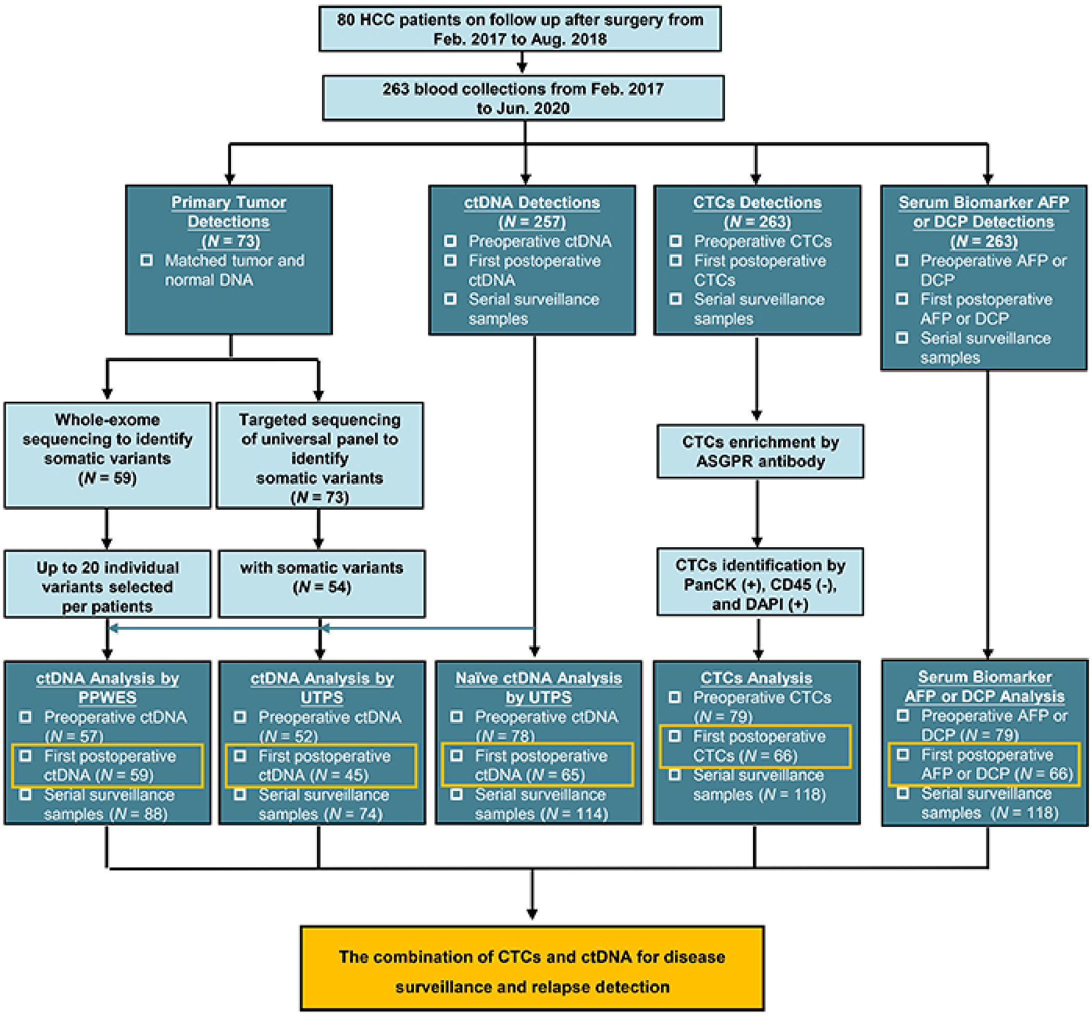

Figure S1. Flow chart for patient enrollment in this study. A total of 80 HCC patients were enrolled and available for follow up in this study from February 2017 to August 2018. The patients underwent hepatectomy (*N* = 59) or liver transplantation (*N* = 21) and started with a follow-up study with median follow-up of 26.1 months (range, 19.3-42.6 months) after surgical therapy. For minimal residual disease (MRD) surveillance, tumor tissues were analysed either by whole exome sequencing (WES) or universal panel targeted sequencing (UPTS) to identify somatic mutations of HCC patients at first. The UPTS composed of HCC driver events was including coding regions of *TP53*, *CTNNB1* and *AXIN1*; promoter regions of *TERT*, and HBV integration regions. The somatic mutations identified in original tumor tissues were used as the basis for ctDNA profiling. Next, ctDNA mutations and CTCs enumeration

in blood samples were analysed dynamically before and after surgery. Moreover, the levels of serum biomarkers (AFP and DCP) and images examinations (CT, MRI and PET-CT) were also monitored in clinic. HCC, hepatocellular carcinoma; cfDNA, cell free DNA; ctDNA, circulating tumor DNA; CTCs, circulating tumor cell; AFP, alpha-fetoprotein; DCP, des-gamma-carboxy prothrombin; CT, computer tomography; MRI, magnetic resonance imaging; PET-CT, positron emission tomography.

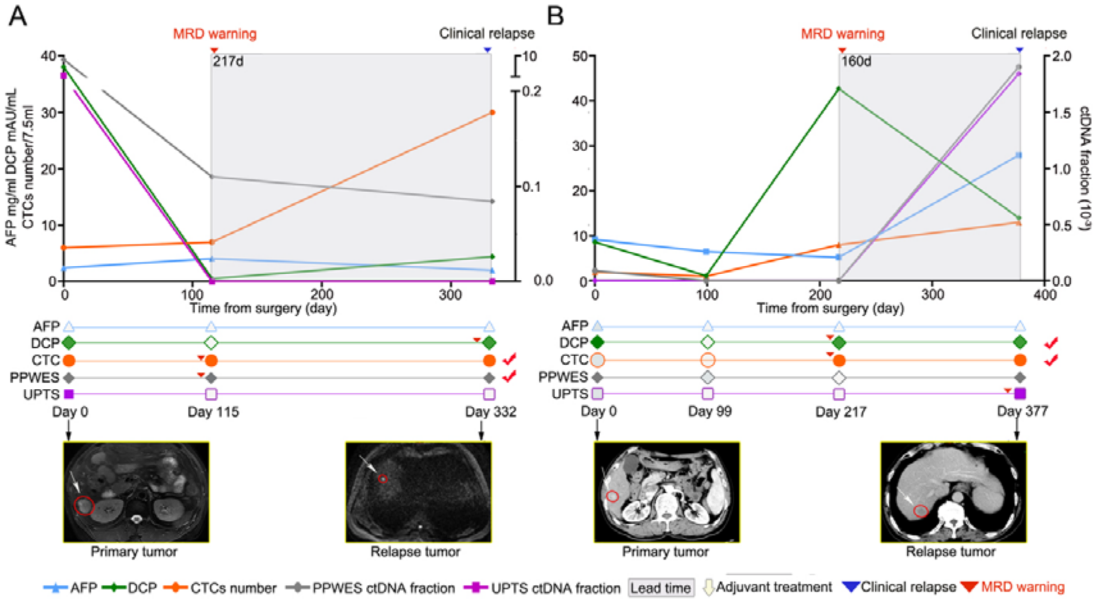

Figure S2. The Dynamic Changes of serum AFP, DCP, ctDNA and CTCs in plasma samples during HCC progression. The upper panels of A and B show the time-course presentation of AFP, DCP, ctDNA and CTC. MRD warning (red triangle) represents the recurrence prediction determined by the reminder of ctDNA (detected by PPWES or UPST assays)/CTCs. Clinical relapse (blue triangle) represents disease recurrence confirmed by imaging diagnosis (CT/MRI/PET-CT). Lead time was indicated by the interval between MRD warning and clinical relapse. The following adjuvant treatments were included: chemotherapy (CTx), radiofrequency ablation (RFA) and radiotherapy (RTx). In the middle panels of A and B, the status of each circulating tumor marker at the time point of continuous follow-up was identified. The solid icon represents the positive state, while the hollow icon represents the negative state. The circulating tumor marker tagged by a red check indicates an effective early warning for recurrence in this case. In the lower panels of A and B, the imaging information from CT/MRI at different time points of each patient is presented.

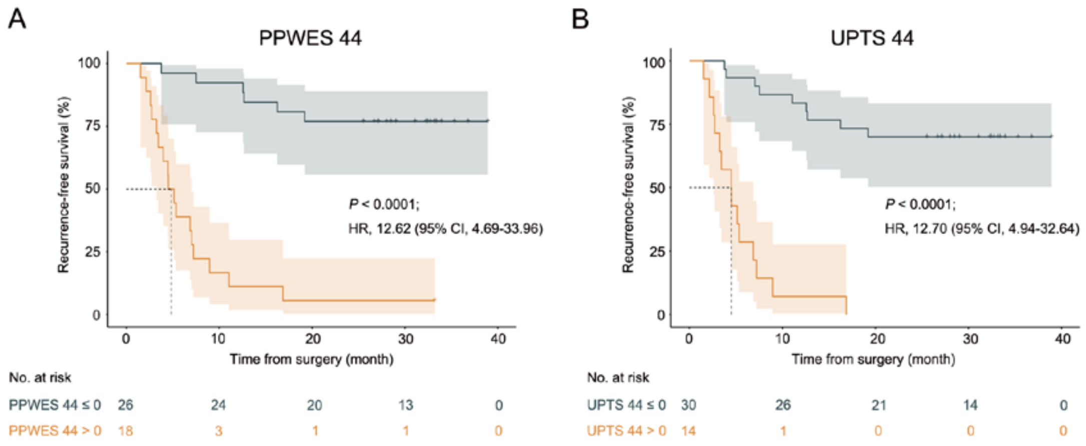

Figure S3. Head-to-head comparison of UPTS and PPWES strategies in predicting recurrence-free survival rates using first post-operative plasma samples. Kaplan–Meier analysis based on (A) ctDNA by UPTS and (B) ctDNA by PPWES in predicting recurrence-free survival rates using the first post-operative plasma sample from 44 HCC patients. The cutoff value, *P* values for log-rank (Mantel–Cox) tests and HR values are indicated in the figure. The dashed line in the figure represents the median survival (months). ctDNA, circulating tumor DNA; UPTS, universal panel targeted sequencing; PPWES, personalized panel targeting mutations from whole-exome sequencing; HR, hazard ratio; CI, confidence interval.

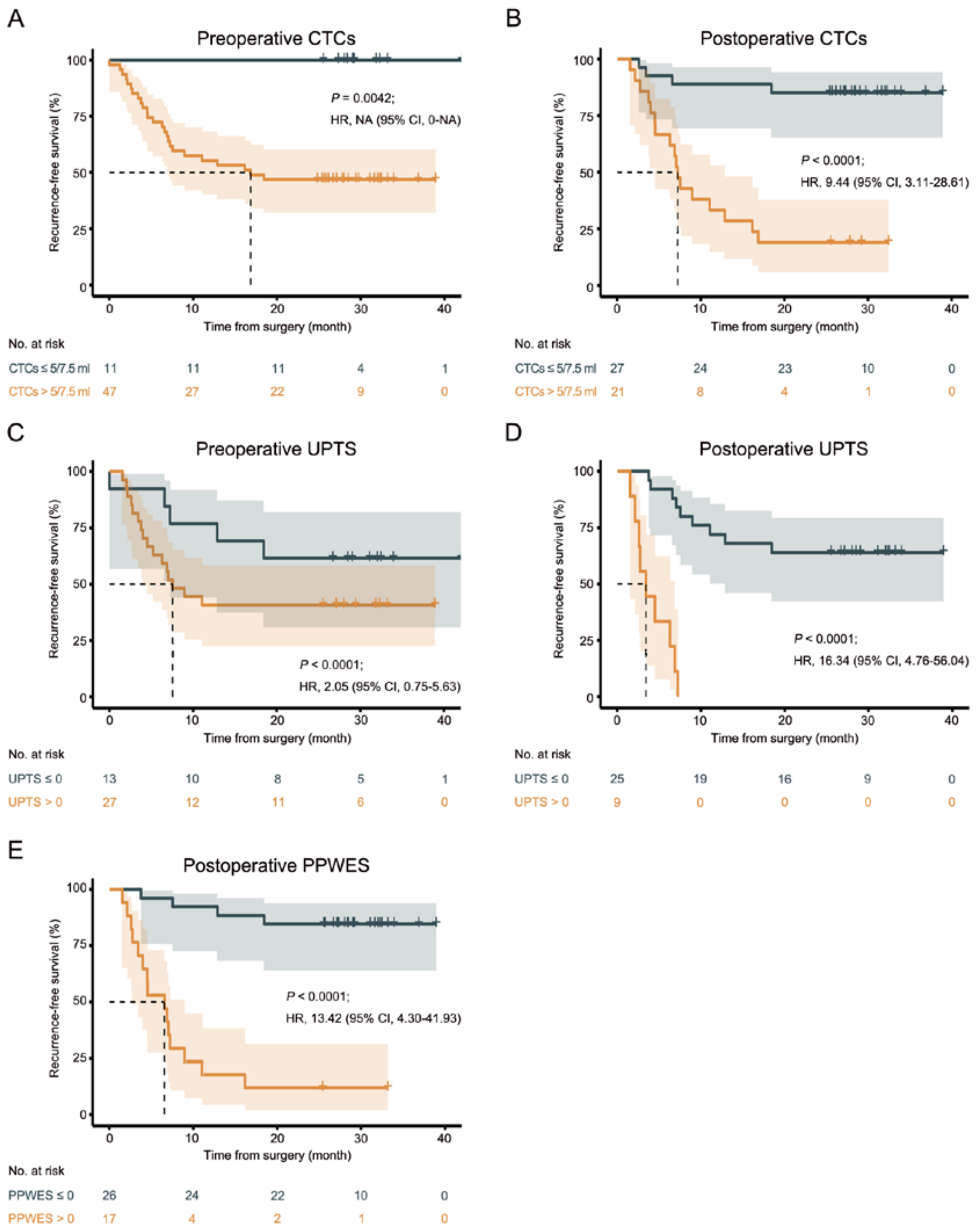

Figure S4. Prediction of recurrence-free survival rates using serum CTCs and ctDNA in the hepatectomy plasma samples. Kaplan–Meieranalysis based on (A) preoperative CTC enumeration, (B) postoperative CTC enumeration, (C) preoperative ctDNA status (ctDNA fraction and HBV integration) by UPTS, (D) postoperative ctDNA status (ctDNA fraction and HBV integration) by UPTS; and (E) postoperative

ctDNA fraction by PPWES. The cutoff value, *P* values from log-rank (Mantel–Cox) tests and HR values are indicated in the figure. The dashed lines in the figure represent the median survival (months). HR, hazard ratio; CI, confidence interval.

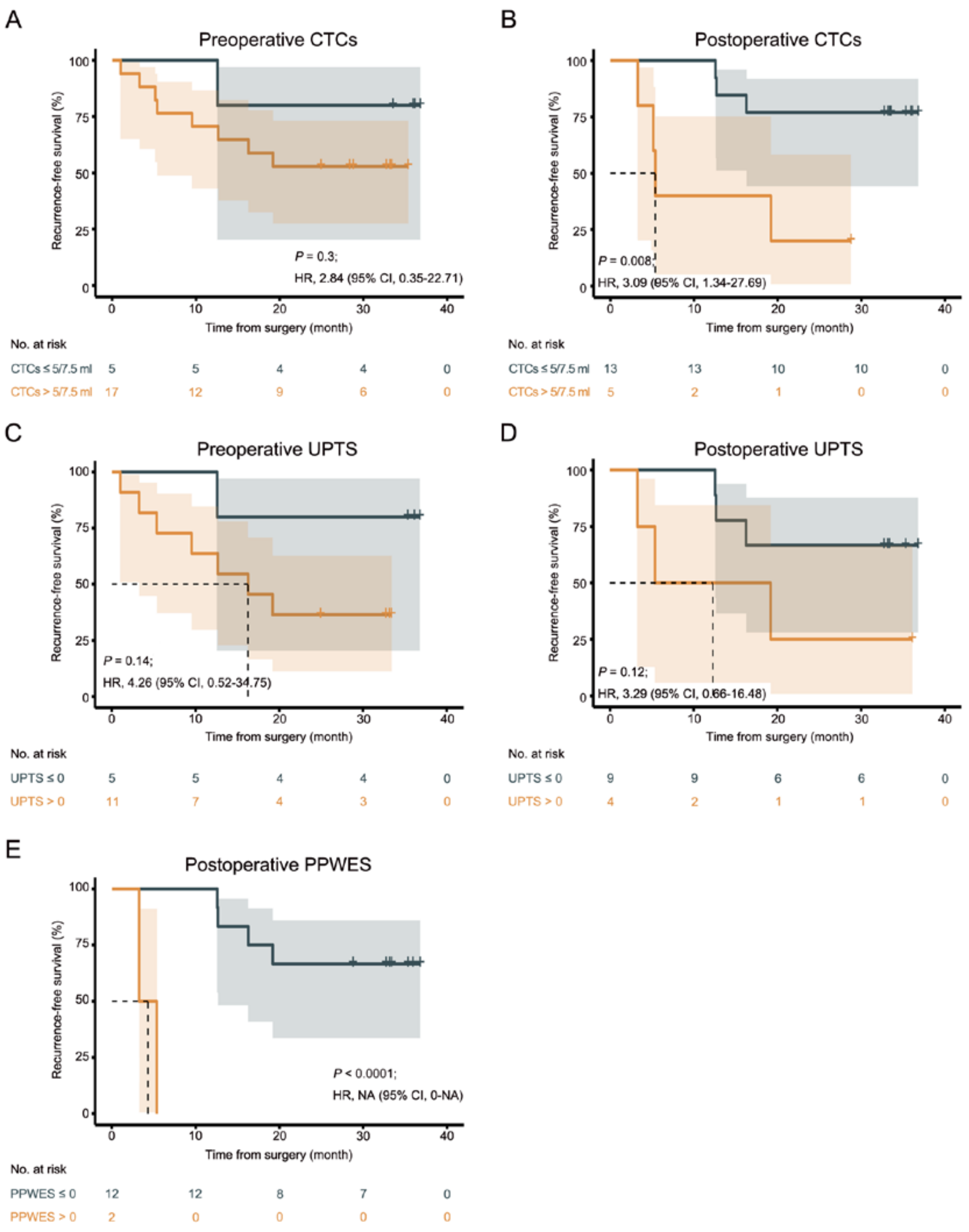

Figure S5. Prediction of recurrence-free survival rates using serum CTCs and ctDNA in the liver transplantation plasma samples. Kaplan–Meier analysis based on (A) preoperative CTC enumeration, (B) postoperative CTC enumeration, (C) preoperative ctDNA status (ctDNA fraction and HBV integration) by UPTS, (D) postoperative ctDNA status (ctDNA fraction and HBV integration) by UPTS; and (E) postoperative ctDNA fraction by PPWES. The cut off value, *P* values from log-rank (Mantel–Cox) tests and HR values are indicated in the figure. The dashed lines in the

figure represent the median survival (months). HR, hazard ratio; CI, confidence interval.

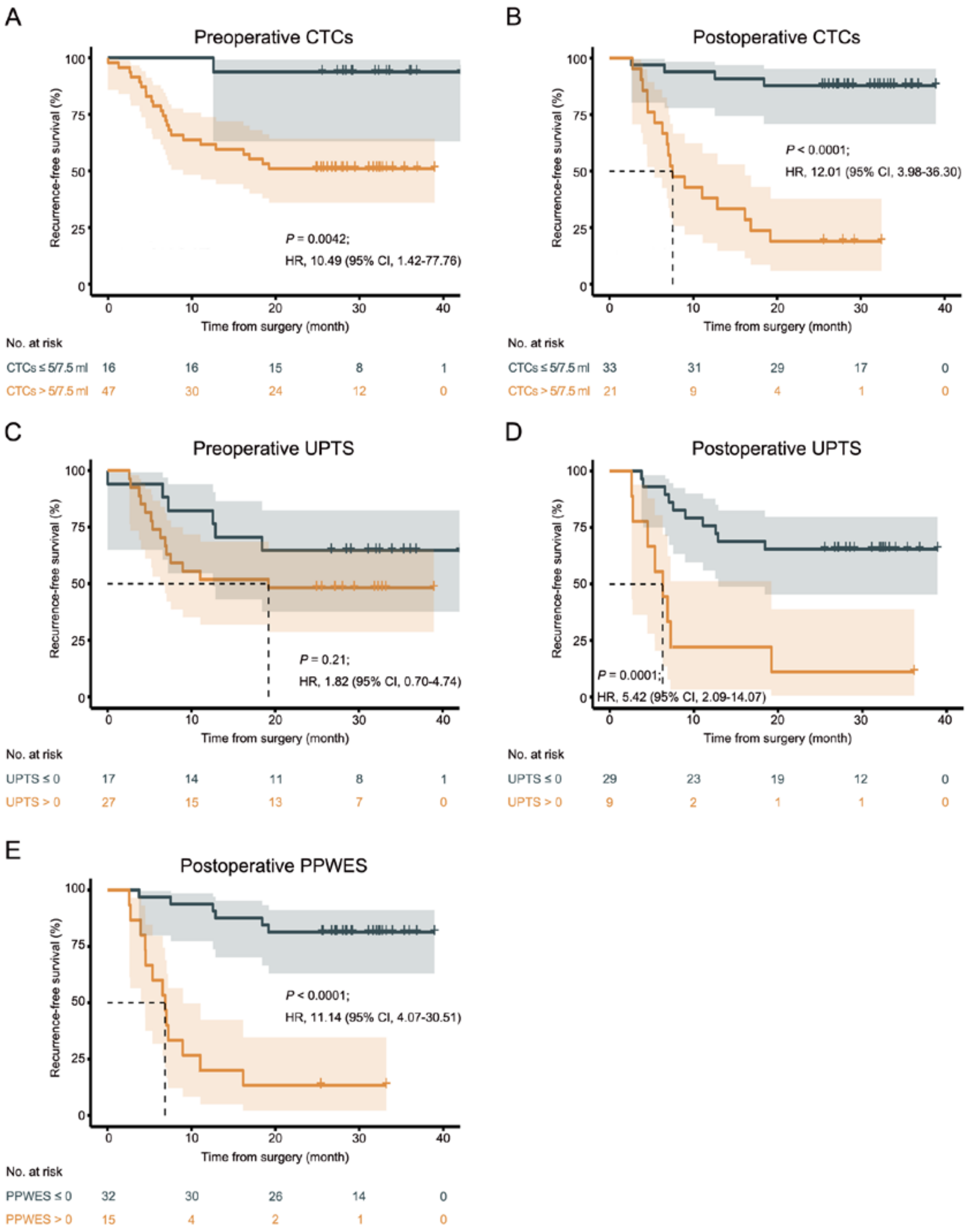

Figure S6. Prediction of recurrence-free survival using serum CTCs and ctDNA in the early stage of HCC patients (AJCC stage = I and II) plasma samples. Kaplan–Meier analysis based on (A) preoperative CTC enumeration, (B) postoperative CTC enumeration, (C) preoperative ctDNA status (ctDNA fraction and HBV integration) by UPTS, (D) postoperative ctDNA status (ctDNA fraction and HBV integration) by UPTS and (E) postoperative ctDNA fraction by PPWES. The cut

off value, *P* values from log-rank (Mantel–Cox) tests and HR values are indicated in the figure. The dashed lines in the figure represent the median survival (months). HR, hazard ratio; CI, confidence interval.

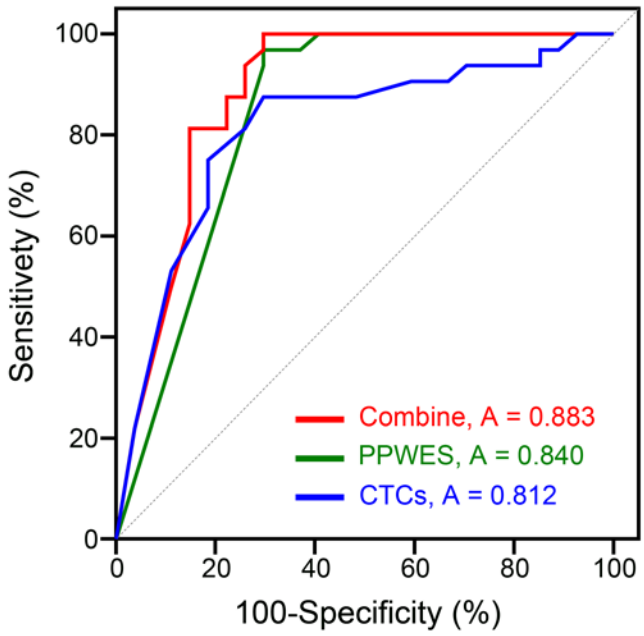

Figure S7. The ROC curves of the serum CTC and ctDNA as a predictive model for HCC recurrence. The capability of CTCs, PPWES and CTCs-ctDNA combine in HCC recurrence prediction was estimated through ROC curves (HCC- recurrence, *N* = 27; non- recurrence, *N* = 32). PPWES, personalized panel targeting mutations from whole-exome sequencing; A, area under curve.

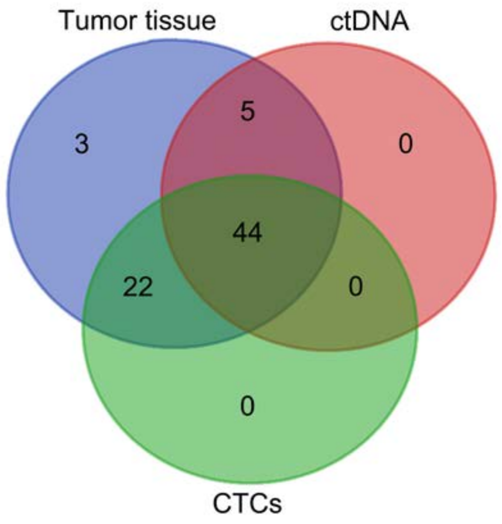

Figure S8. Mutation consistent among tumor tissue, ctDNA and CTCs. Venn diagram shows the consistency of mutations of driver gene (*TP53*, *CTNNB1* and *AXIN1*, *TERT* promoter region) among tumor tissue, ctDNA and CTCs in corresponding cases.
